# An evaluation of the brain distribution of [^11^C]GSK1034702, a muscarinic-1 (M_1_) positive allosteric modulator in the living human brain using positron emission tomography

**DOI:** 10.1186/s13550-014-0066-y

**Published:** 2014-12-05

**Authors:** Khanum Ridler, Vincent Cunningham, Mickael Huiban, Laurent Martarello, Sabina Pampols-Maso, Jan Passchier, Roger N Gunn, Graham Searle, Anissa Abi-Dargham, Mark Slifstein, Jeanette Watson, Marc Laruelle, Eugenii A Rabiner

**Affiliations:** Clinical Imaging Centre, GlaxoSmithKline, Burlington Danes Building, Hammersmith Hospital, Du Cane Road, London, UK; Division of Brain Sciences, Department of Medicine, Imperial College, Burlington Danes Building, Hammersmith Hospital, Du Cane Road, London, UK; Department of Engineering Science, University of Oxford, Parks Road, Oxford, UK; Schizophrenia and Cognitive Disorders Discovery Performance Unit, Neurosciences Centre of Excellence in Drug Discovery, GlaxoSmithKline, New Frontiers Science Park, Harlow, UK; Department of Psychiatry, Columbia University College of Physicians and Surgeons, New York, NY USA; Institute of Psychiatry, PO89, De Crespigny Park, London, SE5 8AF UK; Imanova, Centre for Imaging Sciences, Burlington Danes Building, Hammersmith Hospital, Du Cane Road, London, W12 0NN UK

**Keywords:** GSK1034702, Carbon-11(^11^C), Muscarinic acetylcholine receptor subtype 1 (mAChR), Positron emission tomography (PET), Brain distribution, Biodistribution, M_1_

## Abstract

**Background:**

The ability to quantify the capacity of a central nervous system (CNS) drug to cross the human blood-brain barrier (BBB) provides valuable information for de-risking drug development of new molecules. Here, we present a study, where a suitable positron emission tomography (PET) ligand was not available for the evaluation of a potent muscarinic acetylcholine receptor type-1 (M_1_) allosteric agonist (GSK1034702) in the primate and human brain. Hence, direct radiolabelling of the novel molecule was performed and PET measurements were obtained and combined with *in vitro* equilibrium dialysis assays to enable assessment of BBB transport and estimation of the free brain concentration of GSK1034702 *in vivo*.

**Methods:**

GSK1034702 was radiolabelled with ^11^C, and the brain distribution of [^11^C]GSK1034702 was investigated in two anaesthetised baboons and four healthy male humans. In humans, PET scans were performed (following intravenous injection of [^11^C]GSK1034702) at baseline and after a single oral 5-mg dose of GSK1034702. The *in vitro* brain and plasma protein binding of GSK1034702 was determined across a range of species using equilibrium dialysis.

**Results:**

The distribution of [^11^C]GSK1034702 in the primate brain was homogenous and the whole brain partition coefficient (*V*_T_) was 3.97. In contrast, there was mild regional heterogeneity for GSK1034702 in the human brain. Human whole brain *V*_T_ estimates (4.9) were in broad agreement with primate *V*_T_ and the *f*_P_/*f*_ND_ ratio (3.97 and 2.63, respectively), consistent with transport by passive diffusion across the BBB.

**Conclusion:**

In primate and human PET studies designed to evaluate the transport of a novel M_1_ allosteric agonist (GSK1034702) across the BBB, we have demonstrated good brain uptake and BBB passage consistent with passive diffusion or active influx. These studies discharged some of the perceived development risks for GSK1034702 and provided information to progress the molecule into the next stage of clinical development.

**Trial registration:**

Clinical trial details: ‘Brain Uptake of GSK1034702: a Positron Emission Tomography (PET) Scan Study.’; clinicaltrial.gov identifier: NCT00937846.

**Electronic supplementary material:**

The online version of this article (doi:10.1186/s13550-014-0066-y) contains supplementary material, which is available to authorized users.

## Background

The main goal of early phase drug development is the demonstration of substantial target engagement at doses which are safe and tolerable and the determination of a therapeutic dose range. Positron emission tomography (PET) provides a unique technology for establishing the relationship between the plasma concentration of a compound and the degree of target engagement in tissues such as the brain, which are not accessible by other methods. The method of choice for defining this relationship is the use of a well-understood probe (or radioligand) which allows the quantification of target availability at baseline and following the administration of various doses of a novel drug. This approach allows the construction of a realistic pharmacokinetic model relating the kinetics of a novel drug in blood and plasma to that in tissue and enabling the prediction of target occupancy in future clinical studies [1]. The practical limitation of this approach is the requirement for a well-characterised PET radioligand suitable for the quantification of the target examined. While a range of radioligands are available for the central nervous system (CNS), a large number of targets do not have a suitable radioligand. An alternative, indirect, method to obtain target occupancy information in these cases is to estimate the ‘free’ or unbound concentration of the drug in the tissue of interest (*C*_FT_) by directly labelling the drug molecule. The total concentration of a drug in tissue is of limited utility, as the non-specifically bound fraction is pharmacologically inactive and thus the information is restricted to providing confidence in Blood-brain barrier (BBB) penetration as a binary phenomenon. On the other hand, *C*_FT_ can be combined with an estimate of the affinity of the drug for its target (*K*_D_), obtained from suitable *in vitro* or pre-clinical studies, to obtain an estimate of target occupancy. While such an approach makes several assumptions about the shape of the dose-occupancy relationship and the equivalence between *in vitro*-derived *K*_D_ measures and the true *in vivo K*_D_, it provides a practical method for estimating target occupancy information in the absence of a well-characterised PET radioligand. The theory underpinning this approach has been fully described in a recent manuscript [2]; hence, we present a short summary only.

Passive transport of a drug across the BBB determines that at equilibrium, the *C*_FT_ (given by the product of the total non-displaceable tissue concentration [*C*_ND_] and its free or unbound fraction [*f*_ND_]) will be equal to the free concentration of the drug in the plasma, *C*_FP_ (=*C*_*p*_*f*_*p*_) which implies,

1 $$ {C}_{\mathrm{ND}}\kern0.15em {f}_{\mathrm{ND}}={C}_{\mathrm{p}}\kern0.15em {f}_{\mathrm{p}} $$

The fractional occupancy of the target (Occ_T_) can then be calculated knowing *C*_*p*_*f*_*p*_ and *K*_D_ as,

2 $$ {O}_{{\mathrm{CC}}_{\mathrm{T}}}=\frac{C_{\mathrm{p}}\kern0.15em {f}_{\mathrm{p}}}{C_{\mathrm{p}}\kern0.15em {f}_{\mathrm{p}}+{K}_{\mathrm{D}}} $$

The main goal of a ‘biodistribution’ PET study is thus to test the assumption that the drug crosses the BBB and to assess whether the transport is consistent with passive diffusion. The distribution of a drug in the tissue of interest can be evaluated following its labelling with a radionuclide. The substitution of a ^12^C or ^19^ F atom by the positron emitting isotopes ^11^C or ^18^ F enables the quantification of the drug distribution in tissue over time, without changing its physiochemical or pharmacological characteristics. In principle, one can estimate the total volume of distribution (*V*_T_ [3]), for the drug in the brain, which is equivalent to the equilibrium partition coefficient for the drug between brain and plasma.

If the *V*_T_ of a labelled drug is not reduced following the administration of pharmacologically relevant dose of the same drug, the total brain concentration of the drug (*C*_T_) can be taken to measure the sum of the free and the non-specifically bound components. In this case, the *V*_T_ will be independent of the dose of labelled compound administered and measurements made at tracer concentrations will provide an adequate estimate of drug distribution at pharmacological doses.

3 $$ {V}_{\mathrm{T}}=\frac{C_{\mathrm{T}}}{C_{\mathrm{P}}}=\frac{C_{\mathrm{FT}}}{f_{\mathrm{ND}}}\frac{f_{\mathrm{P}}}{C_{\mathrm{FP}}} $$

It is worth noting that if the specific binding component of a labelled drug is not negligible compared to the free and non-displaceable components, the labelled drug may be used as a radioligand to enable a direct examination of target occupancy.

While *V*_T_, *C*_P_, and *f*P can be measured directly in the course of a PET study in humans, it is not possible to measure *f*ND directly, and hence, *C*_FT_ cannot be obtained without other information being available. If a value for *f*ND can be obtained, then the assumption of passive diffusion across the BBB can be tested. Under the conditions of passive diffusion, at equilibrium, *C*_FT_ = *C*_FP_, and Eq. 3 is simplified to:

4 $$ {V}_{\mathrm{T}}=\frac{f_{\mathrm{P}}}{f_{\mathrm{ND}}} $$

A *V*_T_ measurement from a PET biodistribution study can be compared to the *f*P/*f*ND ratio, and passive diffusion can be assumed if the two are similar. Equilibrium dialysis provides a practical method for measurements of brain tissue *f*ND (and also *f*P if necessary) [2].

We have applied the methodology above to examine the distribution of GSK1034702 in the primate and human brain. GSK1034702 is a selective muscarinic-1 (M1) receptor allosteric agonist, which offers a potential therapy for the treatment of cognitive dysfunction in neurodegenerative disorders. It belongs to the series of novel N-substituted benzimidazolones recently described [4–6].

Muscarinic acetylcholine receptor (mAChR) agonists such as xanomeline have produced some efficacy in the treatment of cognitive dysfunction in patients with Alzheimer's disease (AD) and schizophrenia (SZ) [7,8]. The therapeutic potential of the cholinergic agents tested thus far (cholinesterase inhibitors and muscarinic agonists) is modest and is thought to be limited by peripheral m_2_AChR and/or m_3_AChR-related side effects, such as gastrointestinal disturbances [9].

The discovery of an allosteric (or ectopic) site for m_1_AChR that is not conserved across mAChR subtypes has provided the opportunity to develop m_1_AChR agonists with true receptor selectivity [10]. Allosteric m_1_AChR selective agonists are postulated to display reduced side effects, and thus greater utility, compared with the existing non-selective orthosteric agonists. In an initial study, in healthy smokers using the nicotine abstinence model of cognitive dysfunction, GSK1034702 improved episodic memory [6].

GSK1034702 [11] is a potent allosteric agonist at human recombinant m_1_AChR (pEC_50_ = 8.1 ± 0.1, intrinsic activity (IA) = 0.78 ± 0.02) with 100-fold selectivity over human m_2-5_AChR receptors and with >90 over other molecular targets from a variety of classes. GSK1034702 is a weak substrate for P-glycoprotein (PGP) (efflux ratio of 2.6:1 in MDCK cell line expressing human MDR1) and has displayed some species variability in brain-to-plasma ratios (brain/blood ratios of 0.4, 0.6 and 2.0:1, in mouse, rat and marmoset, respectively; GlaxoSmithKline (GSK) data on file). Therefore, the delivery of GSK1034702 into human brain could have been adversely affected. Initially, the primate study was carried out in order to demonstrate that [^11^C]GSK1034702 crossed the BBB in primates, before proceeding to expensive human studies. The human study aimed to use PET with radioactively labelled GSK1034702 to help ascertain the role PGP plays in limiting brain penetration in the presence and absence of a pharmacological relevant oral non-labelled dose of 5 mg.

Here, we investigated the distribution of GSK1034702 in the living human brain. These data provide vital information to determine whether GSK1034702 can be used at doses that offer therapeutic benefit to patients with AD and SZ without inducing intolerable muscarinic-related side effects.

## Methods

### Radiosynthesis of [^11^C]GSK1034702

**Figure 1:**
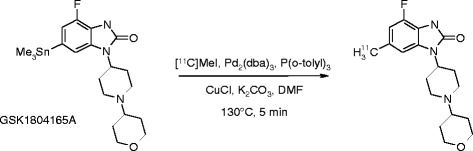
**Synthesis route for [** ^**11**^ **C]GSK1034702.**

[^11^C]GSK1034702 was prepared as described previously [5] via a palladium-catalysed cross coupling reaction between GSK1804165A and [^11^C]CH_3_I (Figure 1).

### Evaluation of [^11^C]GSK1034702 kinetics in the *Papio anubis* brain

#### Study design

The brain distribution of carbon-11-labelled GSK1034702 ([^11^C]GSK1034702) was conducted in two anaesthetised baboons (*P. anubis*), to assess its transport across the BBB. These studies were conducted at Columbia University Medical Center, New York. The study was approved by the Columbia University Institutional Animal Care and Use Committee (IACUC).

Two adult baboons were studied over a 6-week period. Subject A (an ovariectomised female) received two injections of [^11^C]GSK1034702 22 days apart, while subject B (male) received a single injection of [^11^C]GSK1034702. Each experimental session included a single dynamic PET scan.

#### Data acquisition

Fasted animals were immobilised with intramuscular ketamine injection (10 mg/kg), and anesthetised with 1.8% isoflurane via an endotracheal tube. Vital signs were monitored every 10 min and the temperature was kept constant at 37°C with heated water blankets. An intravenous (IV) perfusion line was used for hydration and injection of the radiotracer. A catheter was inserted in a femoral artery for arterial blood sampling. The head was positioned at the centre of the field of view. PET imaging was performed with the ECAT EXACT HR + PET scanner (Siemens/CTI, Knoxville, TN) operating in 3D mode (in-plane resolution of 4.3, 4.5, 5.4 and 8.0 mm, full width at half maximum (FWHM) at a distance of 0, 1, 10 and 20 cm from the centre of the field of view) [12]. A 15-min transmission scan was obtained prior to radiotracer injection for attenuation correction. The injected mass and radioactivity had an upper limit of 1.16 μg and 47 MBq, respectively. Following IV administration (45-s bolus) of a single dose of [^11^C]GSK1034702 dynamic PET, arterial blood and metabolite data were acquired for 120 min (subject A) for the first two scans and only 60 min for the third scan due to a technical issue (subject B).

#### Image processing and quantification of binding parameters

Regions of interest (ROIs) were drawn on previously acquired T1-weighted anatomical magnetic resonance imaging (MRI) brain images for each animal. The following regions were defined: cerebellum, hippocampus, thalamus, striatum, frontal cortex and occipital cortex. Regions were drawn in the MEDx (Sensor Systems Inc., Sterling, VA, USA) software environment. PET emission data were attenuation-corrected using the transmission scan, and frames were reconstructed using a Shepp filter (cutoff 0.5 cycles/projection rays). Decay-corrected PET images were registered to the MRI images by maximisation of mutual information using SPM2 and Matlab software. Regional boundaries were transferred from the MRI to the individual registered PET frames, and time-activity curves were measured in the MEDx environment. Right and left regions were averaged. For a given animal, the same regions were used for all scans.

For all non-human primate studies, the non-protein-bound fraction of [^11^C]GSK1034702 in arterial plasma (*f*_P_) was quantified using the following methodology: triplicate 200 μL aliquots of plasma collected before injection were mixed with [^11^C]GSK1034702, pipetted into ultrafiltration units (Centrifree; Amicon, Danvers, MA, USA) and centrifuged at room temperature (20 min at 4,000 rpm). At the end of centrifugation, plasma and ultrafiltrate activities were counted and *f*_P_ was calculated as the ratio of ultrafiltrate to total activity concentrations.

Brain time activity curves (TACs) were corrected for the contribution of blood activity assuming a 5% blood volume. A metabolite-corrected arterial input function and regional TACs were fitted to a compartmental model to derive regional *V*_T_ estimates as well as a whole brain *V*_T_.

### Evaluation of [^11^C]GSK1034702 kinetics in the human brain

#### Study design

An open-label PET study (GSK study number 110771) was conducted to assess the transport of [^11^C]GSK1034702 across the BBB and its distribution in the living human brain. Four healthy males aged between 35 and 55 years old, with body mass index (BMI) between 19.0 and 29.0 kg/m^2^, and the absence of past or present neurological, medical or psychiatric illnesses and concomitant medications were enrolled. Clinical status was assessed by history, physical examination, routine blood tests, urine toxicology and electrocardiogram. All subjects provided written informed consent. The study was approved by the Edinburgh Independent Ethics Committee for Medical Research and permission to administer radioisotopes was obtained from the Administration of Radioactive Substances Advisory Committee of the UK. Subjects were recruited by and monitored by the Hammersmith Medicines Research (HMR) clinic. All scanning procedures were performed at the GlaxoSmithKline Clinical Imaging Center, Hammersmith Hospital, London.

Each subject underwent a single study day, during which PET imaging was performed. T1-weighted structural MRI scan was performed for all subjects to provide neuroanatomical information. A tracer dose of [^11^C]GSK1034702 (mass <50 μg) was administered as an IV bolus immediately before each PET scan. During the scanning day, each subject received a baseline [^11^C]GSK1034702 scan, followed by a single 5-mg oral dose (p.o.) of GSK10347012 and a subsequent [^11^C]GSK1034702 scan approximately 4 h post-dose. Subjects returned to the clinic for a follow-up visit 7 to 14 days following the oral dose of GSK1034702.

#### Data acquisition

Dynamic PET data were acquired using a Siemens Biograph 6 Truepoint with TrueV PET-CT (Siemens Healthcare, Malvern, PA, USA) in three-dimensional (3D) mode. A computed tomography (CT) scan was acquired for attenuation correction of PET data prior to administration of the radiotracer. A cannula was inserted under local anaesthesia into the radial artery, contralateral to the tracer injection site, and used to collect arterial blood samples to estimate the time course of radioactivity and metabolism of GSK1034702. The radiotracer was administered as an IV bolus over 20 s, and dynamic PET data were acquired for 90 min. The injected mass of GSK1034702 did not exceed 50 μg.

Arterial blood radioactivity was measured continuously for the first 15 min after [^11^C]GSK1034702 injection, using a continuous sampling system (ABSS Allogg, Mariefred, Sweden). In addition, 12 serial discrete blood samples were obtained (at 5, 10, 15, 20, 25, 30, 40, 50, 60, 70, 80 and 90 min post injection), to determine whole blood and plasma activity. Seven samples (at 5, 10, 20, 30, 50, 70 and 90 min) were further processed by HPLC to measure the fraction of plasma activity representing unmetabolised GSK1034702. For all human studies, the non-protein-bound fraction of [^11^C]GSK1034702 in arterial plasma (*f*_P_) was assessed by ultrafiltration for the blood sample taken at the beginning of the scan.

High-resolution (HR) 3D volumetric MRI scans (MPRAGE sequence: TR = 3,000 ms, TE = 3.66 ms, flip angle = 9°, voxel size = 1 mm^3^, 208 slices) were acquired on a 3 T Tim Trio MRI scanner system (Siemens Healthcare) to provide a T1-weighted image for co-registration with their PET data.

Serial venous blood samples were obtained at 0, 1, 2, 3, 4 and 5.5 h after the administration of a single oral dose of 5 mg of GSK1034702, to estimate the time course of GSK1034702 plasma concentration. Plasma samples were analysed using a validated analytical method.

#### Image processing and quantification of binding parameters

Emission PET data were binned into 26 frames (durations: 8 × 15 s, 3 × 60 s, 5 × 2 min, 5 × min, 5 × 10 min) and reconstructed using Fourier re-binning and a two-dimensional (2D) filtered back projection algorithm with a ramp filter [13]. Image data were then smoothed with a Gaussian filter (5 mm FWHM). The PET reconstruction process included corrections for scatter, randoms and attenuation [13]. Dynamic PET data were corrected for motion via frame-to-frame image registration and aligned with the structural T1 MRI image using SPM5 (Wellcome Trust Centre for Neuroimaging, http://www.fil.ion.ucl.ac.uk/spm) with a mutual information cost function.

The CIC Neuroanatomical Atlas [14] was non-linearly deformed into the individual's space to obtain a personalised anatomical parcellation of ROIs. These ROIs were applied to the dynamic emission data, and regional TACs were obtained for the whole brain, frontal cortex, occipital cortex, striatum, thalamus, hippocampus and cerebellum.

In addition, as some degree of regional heterogeneity was observed in the binding of [^11^C]GSK1034702 in the human brain, a regular-sized rectangle (432 mm^3^) was manually placed over the area of highest signal in the medial temporal lobe (MTL), centred in the hippocampus for each subject and applied to the dynamic emission data to generate a MTL TAC. See Additional file 1: Figure S1A, for further methological details of ROI definition.

Regional TACs with an arterial plasma metabolite- corrected input function were fitted using compartmental analyses, and estimates of the delivery rate constant for transfer from arterial plasma to tissue (*K*_1_) and *V*_T_ were obtained. *V*_T_ estimates derived were compared to *in vitro* estimates derived from equilibrium dialysis to investigate the BBB transport process.

The kinetic parameters from the PET studies were applied to the pharmacokinetic (PK) data from the individual subjects in order to estimate the regional distribution of GSK103702 following an oral dose.

### Brain and plasma protein binding of GSK1034702

The *in vitro* brain and plasma protein binding of GSK1034702 across a range of species was determined using equilibrium dialysis as described previously [2].

## Results and discussion

### Evaluation of [^11^C]GSK1034702 CNS penetration and kinetics in the *P. anubis* brain

#### Summary scan data

The injected dose of [^11^C]GSK1034702 was 27.7 ± 17.7 MBq (with these and subsequent values expressed as mean ± standard deviation [SD]), with the average injected mass of 1.05 ± 0.05 μg. The free fraction of [^11^C]GSK1034702 in plasma (*f*P) was 0.54 ± 0.21.

#### Quantification of PET data

A two-tissue compartmental model was identified as the most appropriate kinetic model based on parsimony criteria and was used to derive regional *V*_T_ values. The distribution of [^11^C]GSK1034702 was homogenous throughout the brain, with regional *V*_T_ values of similar magnitude (see Table 1 and associated *K*_1_ values are reported in Additional file 2: Table S1A), suggesting that any specific binding component of [^11^C]GSK1034702 is small compared to the ‘free and non-specific’ binding component. The whole brain *V*_T_ for [^11^C]GSK1034702 in the *P. anubis* brain was 3.97 ± 0.38. There was high uptake in the pituitary which is situated outside the BBB.

**Table 1:** **Regional** ***V***_**T**_ **values in** ***Papio anubis*** **brain**

**ROI**	**Subject A Exam 1**	**Subject A Exam 2**	**Subject B Exam 1**
Cerebellum	4.07	3.79	3.09
Hippocampus	4.78	4.62	3.52
Thalamus	4.06	3.91	2.75
Striatum	3.86	3.95	3.21
Frontal Ctx	3.92	3.73	3.52
Occipital Ctx	4.49	4.64	3.89
Whole brain	4.20	4.19	3.52

*V*_T_ = volume of distribution (mL.cm^−3^).

### Evaluation of [^11^C]GSK1034702 CNS penetration and kinetics in the human brain

#### Summary scan data

A total of four male subjects, with a mean (±SD) age of 43.5 ± 5.1 years and a mean (±SD) BMI of 26.6 ± 2.9 kg/m^2^, were enrolled into the study. In all, five technically adequate data sets were obtained in three subjects (three baseline scans and two scans following the administration of oral GSK1034702). The post-dose scan for subject 1 was not performed due to the failure of [^11^C]GSK1034702 synthesis, while the acquisition of arterial blood data for both scans of subject 3 was compromised by problems with a blood sampling device, and hence, a quantitative tracer kinetic analysis of the scans from this subject was not performed.

The mean (±SD) injected dose of [^11^C]GSK1034702 was 308 ± 26 MBq, with a mean (±SD) injected GSK1034702 mass of 42.9 ± 5.2 μg.

A single oral dose (p.o.) of 5 mg GSK1034702 was well tolerated. One subject experienced two mild and transient adverse events during this study (salivary hypersecretion and dysgeusia). Both adverse events were considered to be drug-related. No serious adverse events were observed. The plasma concentration of GSK1034702 following the administration of 5 mg p.o, and the measured free fraction in the plasma are presented in Table 2.

**Table 2:** **GSK1034702 plasma concentration at start of PET scan and GSK1034702 plasma free fraction (** ***f***_**P**_ **)**

			***f***_**P**_	
	**Time between oral dose of GSK1034702 and start of post-dose PET scan (hours)**	**GSK1034702 plasma concentration at start of post-dose PET scan (ng/mL)**	**Baseline**	**Post-dose**
Subject 1	-	-	0.42	-
Subject 2	3.2	7.9	0.43	0.41
Subject 3	4.4	13.2	0.43	0.43
Subject 4	3.8	10.4	0.51	0.48
Average			0.45	0.44

The dose for all subjects was 5 mg of GSK1034702. Time given is the time in hours post oral dose of GSK1034702 at which the PET scan acquisition started. GSK1034702 PK concentrations were taken between results from the start of the scan are taken between 4 and 50 min before the PET scan. *In vivo* plasma-free fractions were measured from plasma samples acquired from the four subjects during each PET scan. *f*_P_ = plasma-free fraction.

#### Quantification of PET data

**Figure 2:**
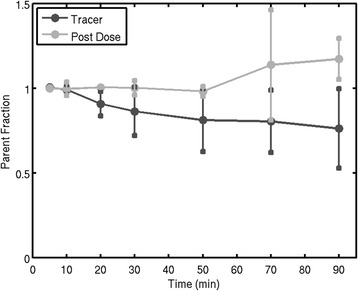
**Parent fraction of [** ^**11**^ **C]GSK1034702 over the time course of the PET scan for all subjects (** ***N*** **= 4).** Error bars denote the Standard Deviation of the [^11^C]GSK1034702 parent fraction.

**Figure 3:**
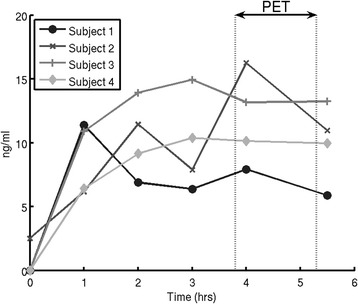
**Clinical Study plasma concentration of GSK1034702 over time (in h).** Subjects were scanned between 3 and 5 h after a single oral dose of GSK1034702. Figure 3 shows that PK levels of GSK1034702 had reached a peak in all subjects prior to the acquisition of the PET data.

[^11^C]GSK1034702 underwent minimal metabolism over the time course of the PET scan during both the baseline and the post-dose sessions (Figure 2). Following the administration of oral GSK10347012, the plasma concentration reached peak levels by the time of the post-dose scan in all subjects and remained relatively constant over the course of the PET scan (Figure 3). The plasma concentrations achieved were consistent with the prediction based on the First-Time-In-Human safety and tolerability study (GSK study number 110623 and study number NCT00743405 on CT.gov).

**Figure 4:**
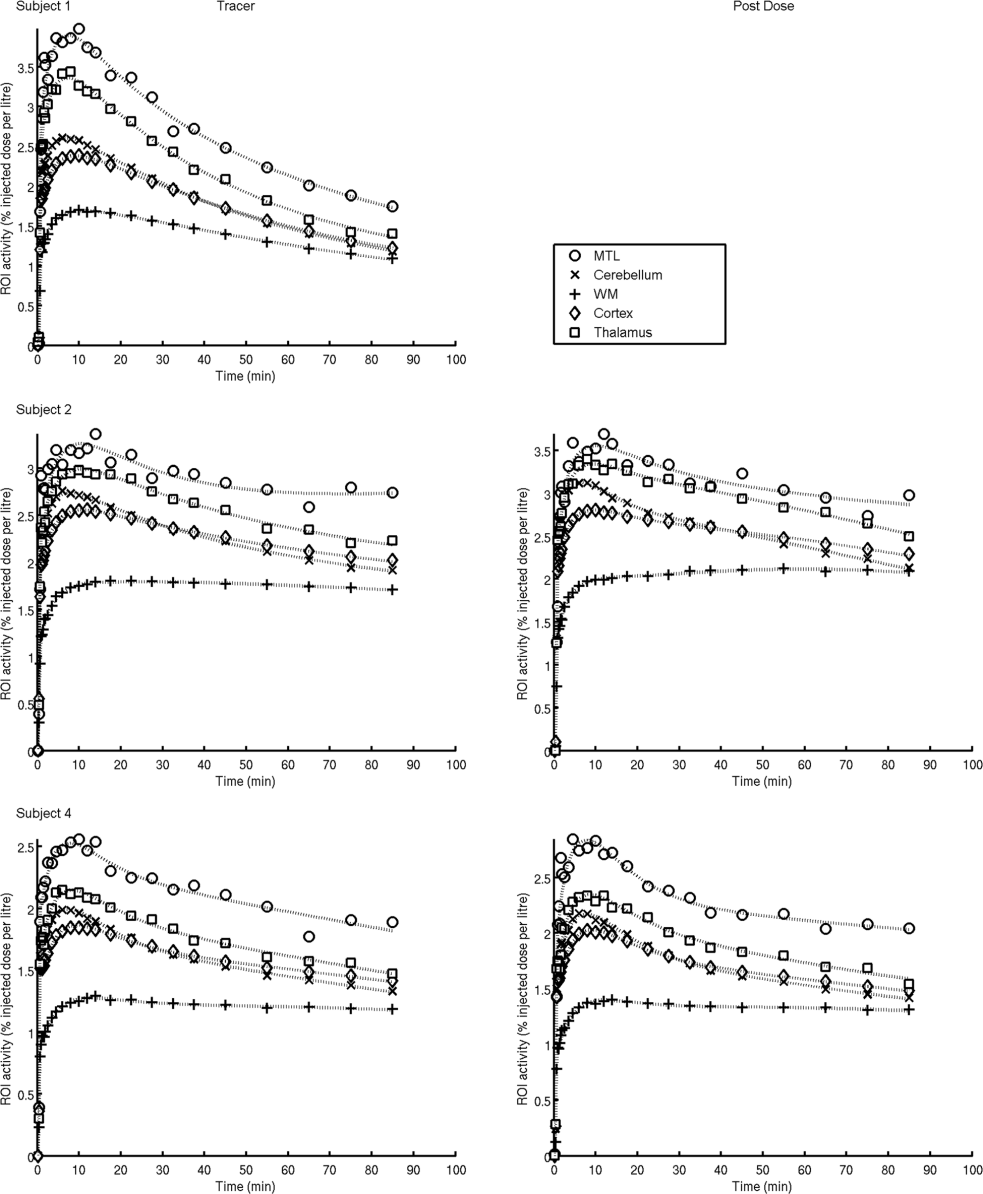
**Regional time activity curves of [** ^**11**^ **C]GSK1034702 in the human brain and model fits.** Regions of interest: white circle, MTL = medial temporal lobe; x, cerebellum; white square, thalamus; diamond, cortex = sum of cortical regions including both cortical grey and white matter; plus sign, WM = all cerebral white matter. Solid lines are two tissue compartmental model fits. For each subject, a regular-sized rectangle (432 mm^3^) was manually placed over the area of highest signal in the medial temporal lobe (MTL), centred in the hippocampus; this is presented in the data as the MTL ROI.

**Figure 5:**
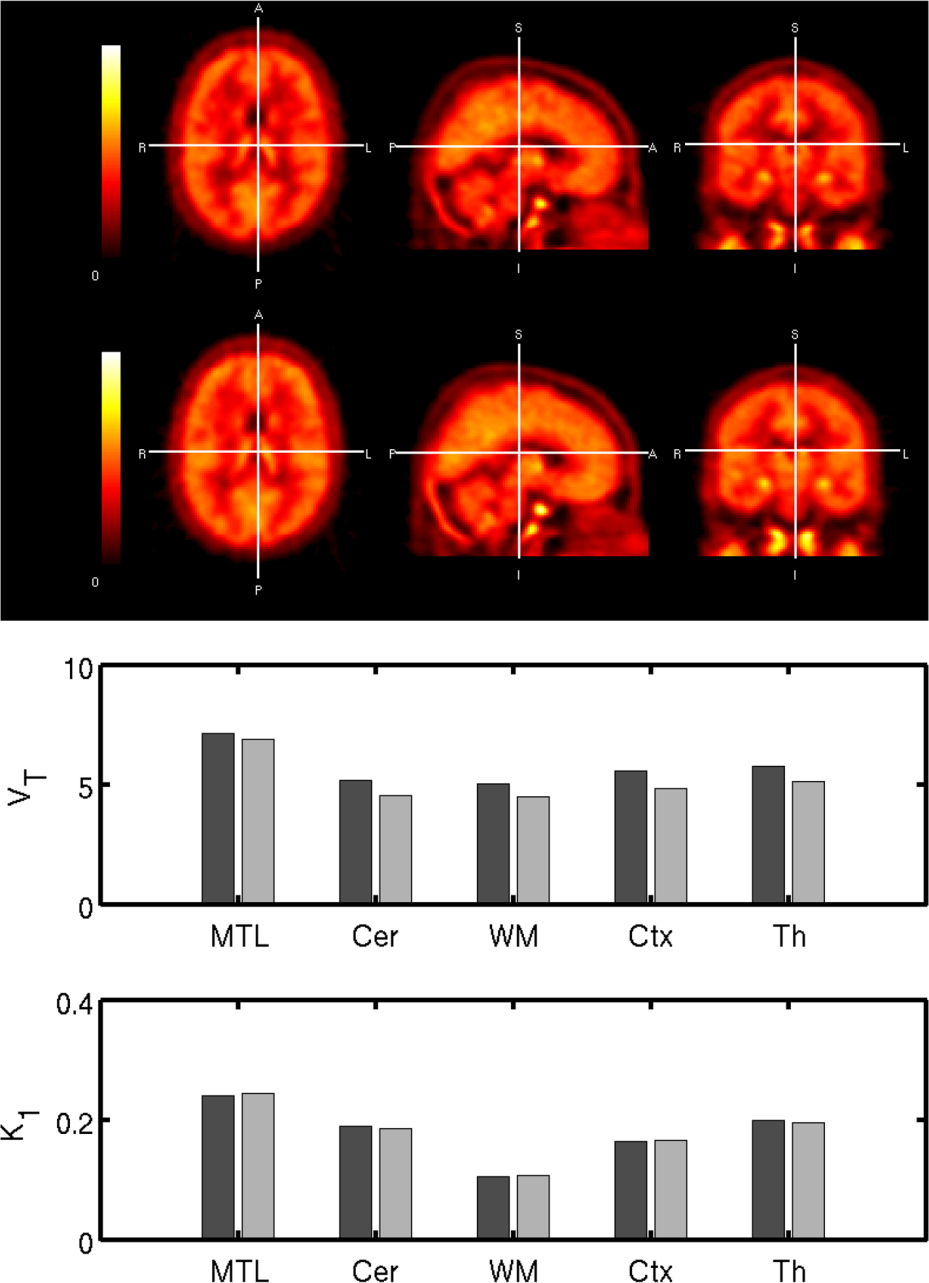
**[** ^**11**^ **C]GSK1034702 in Subject 4 in the human brain.** Tracer [^11^C]GSK1034702 PET data - integral images 0-120 min in subject 4. Post dose 5 mg of GSK1034702 [^11^C]GSK1034702 PET data - integral images 0-120 min in subject 4. *K*_1_ = rate constant for transfer from arterial plasma to tissue (mL.cm^−3^.min^−1^). *V*_T_ = volume of distribution (mL.cm^−3^). Regions of interest: MTL = medial temporal lobe (ROI defined above), cerebellum, thalamus and striatum and cortex = sum of cortical regions (includes grey and white matter); white matter = all cerebral white matter.

The regional brain TACs for all scans analysed are presented in Figure 4 and distribution by a representative subjects integral image in Figure 5. A two-tissue compartmental model was identified as the most appropriate kinetic model based on parsimony criteria, which employed the Akaike information criteria [15] for model selection, and was used to derive regional *V*_T_ values (Table 2).

As opposed to the non-human primate data, there appeared to be some regional heterogeneity in the distribution of [^11^C]GSK1034702, with the *V*_T_ in the MTL in the range of 7-13, while that in the white matter being in the range of 4-5. The whole brain V_T_ in humans was 4.9. We did not expect to see displaceable [^11^C]GSK1034702 binding, and hence did not expect a change in binding following the administration of an oral dose of 5 mg GSK1034702. As expected, we saw no significant change in either V_T_ or K_1_ for [^11^C]GSK1034702, however, there was a non-significant reduction in the MTL area in both subjects where following administration of 5 mg of GSK1034702 (see Table 3 and Figures 4 and 5).

**Table 3:** **Regional** ***V***_**T**_ **,** ***K***_**1**_ **values and GSK1034702 injected mass (μg) and [** ^**11**^ **C]GSK1034702 injected dose in MBq for each scan in the human brain**

**ROI**	**Subject 1**		**Subject 2**				**Subject 4**			
	**Scan 1 (baseline)**		**Scan 1 (baseline)**		**Scan 2 (post dose)**		**Scan 1(baseline)**		**Scan 2 (post dose)**	
	***K***_**1**_	***V***_**T**_	***K***_**1**_	***V***_**T**_	***K***_**1**_	***V***_**T**_	***K***_**1**_	***V***_**T**_	***K***_**1**_	***V***_**T**_
MTL	0.33	7.81	0.19	13.3	0.16	8.30	0.24	7.11	0.25	6.91
Cerebellum	0.23	5.33	0.19	4.75	0.15	3.69	0.19	5.17	0.19	4.56
White matter	0.13	4.63	0.10	4.38	0.08	4.01	0.11	5.04	0.11	4.49
Cortex	0.19	5.37	0.16	5.00	0.12	4.03	0.16	5.57	0.17	4.81
Striatum	0.20	5.59	0.16	5.05	0.13	5.29	0.18	5.83	0.18	4.93
Thalamus	0.29	6.29	0.19	5.44	0.15	4.39	0.20	5.74	0.20	5.11
GSK1034702 injected mass	38.48		34.17		49.35		46.02		44.19	
[^11^C]GSK1034702 injected dose	284.19		322.12		263.02		300.50		325.50	

Regions of interest: MTL = medial temporal lobe (ROI defined above), cerebellum, thalamus and striatum and cortex = sum of cortical regions (includes grey and white matter); white matter = all cerebral white matter. *K*_1_ = rate constant for transfer from arterial plasma to tissue (mL.cm^−3^.min^−1^). *V*_T_ = volume of distribution (mL.cm^−3^). GSK1034702 injected mass in microgrammes. [^11^C]GSK1034702 injected dose in megabecquerel.

#### Brain kinetics of GSK1034702 in the human brain

**Figure 6:**
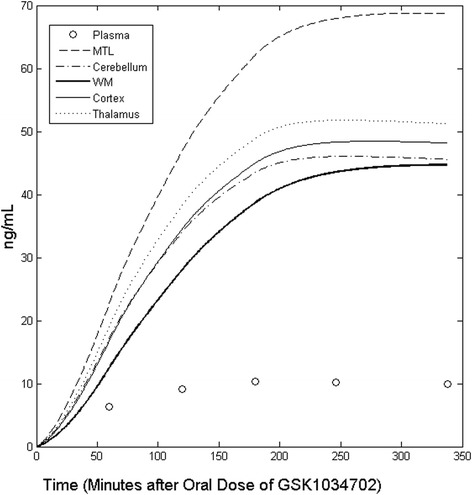
**Predicted time course of GSK1034702 concentration in the human brain following a single 5-mg oral dose in subject 4.** Regions of interest: MTL = medial temporal lobe (defined above), WM = all cerebral white matter, cortex = sum of cortical regions (including grey and white matter), plasma = the measured plasma concentration of GSK1034702 for comparison.

The kinetic parameters derived from the two-tissue kinetic model were applied to the plasma pharmacokinetic data from individual subjects in order to estimate the regional pharmacological GSK1034702 concentration time course following an oral dose. These data predict a peak brain concentration only slightly later than that in the plasma, indicative of a rapid exchange of GSK1034702 across the BBB (see Figure 6 for illustrative results from subject 4).

### Estimation of GSK1034702 transport across the BBB

The *in vitro* plasma protein and brain tissue binding of GSK1034702 were determined in mouse, rat, dog, marmoset, monkey and human tissue by equilibrium dialysis. The bound fractions in plasma and brain were similar in all species tested (Table 4).

**Table 4:** ***In vitro*** **plasma protein binding and brain tissue binding of GSK1034702**

**Species**	**Plasma protein binding (%)**	***f***_**P**_	**Brain tissue binding (%)**	***f***_**ND**_
Mouse	48.2 (±1.2)	0.52	86.7 (±0.6)	0.13
Rat (CD)	47.2 (±1.4)	0.53	87.7 (±0.5)	0.12
Rat (LH)	45.5 (±1.5)	0.55	84.9 (±1.0)	0.15
Dog	44.5 (±3.6)	0.56	81.8 (±1.8)	0.18
Monkey	41.7 (±2.7)	0.58	83.2 (±1.1)	0.17
Human	55.8 (±3.6)	0.44	N/A	

Plasma protein binding and brain tissue binding of GSK1034702 evaluated by equilibrium dialysis over 5 h at concentration of 1 μg/mL. Values represent mean ± standard deviation from *n* = 6 determinations, *f*_P_ = plasma-free fraction, *f*_ND_ = brain-free fraction. CD = Sprague Dawley rat and LH = Lister hooded rat.

The *in vitro* equilibrium dialysis *f*_P_ values for the monkey and human plasma (0.58 and 0.44, respectively) were consistent with the values obtained by ultrafiltration in the course of the PET studies in baboon and human plasma *in vivo* (0.54 and 0.45, respectively). Monkey brain *f*_ND_ (=0.17) data was used for both human and baboon estimation.

The *f*_P_/*f*_ND_ ratios derived from the equilibrium dialysis estimates indicated that if GSK1034702 crosses the BBB by passive diffusion, the expected *V*_T_ would be 3.4 and 2.6 in the primate and human brain, respectively. The *V*_T_ measured in the primate and human cortex (4.0 and 4.9) is in broad agreement with a passive diffusion model.

## Discussion

The exploding costs of drug development, and in particular in the neurosciences, make it imperative that decisions about compound progression are made as early as possible, in order to terminate unsuitable candidates before they reach later, more expensive phases of development. The fundamental requirement of any new molecule is that it is able to reach its site of action in pharmacologically meaningful quantities, at doses which are generally safe and tolerable. The quantification of target engagement in the CNS is hampered by our inability to directly assay the target in the living human brain and the frequent species differences encountered. The direct quantification of drug interaction with its target via the use of PET with a selective radioligand is the method of choice in deriving the relationship between the plasma concentration of a compound and the occupancy of its target. This technique can provide information on target occupancy following single-dose administration that can be extrapolated to provide accurate estimates of occupancy in the clinically meaningful setting of repeat-dose administration [1].

A large fraction of CNS targets of interest are not suitable for examination by this method, due to the lack of suitable radioligands. In such situations, the traditional approach has been to label the molecule of interest and derive the partition coefficient between the labelled drug in plasma and in the brain, as a semi-quantitative measure of drug availability at the target. Such measures are useful, but more limited than direct estimates of target occupancy, since the partition coefficient (known in the PET literature as the total volume of distribution, *V*_T_) provides quantification of the total concentration of the drug in the brain. The total concentration is composed of the drug specifically bound to the target (*V*_S_) and the non-displaceably bound drug (*V*_ND_), which comprises drug-bound non-specifically as well as the free drug. If the *V*_S_ can be estimated robustly from kinetic data, direct target engagement may be evaluated, using the methods developed for target-specific radioligands. However, this situation is rare for drugs in development, primarily due to the presence of high levels of non-specific binding (high *V*_ND_). Hence, for a large proportion of labelled drugs, the measured *V*_T_ is essentially equivalent to *V*_ND_.

The pharmacologically relevant fraction of the drug is the free concentration, *C*_FT_, which is able to interact with the target, and the *f*_ND_ is needed to estimate *C*_FT_ when only the total tissue concentration is available. If the drug transport across the BBB is by passive diffusion, then at equilibrium, the free concentration of the drug in plasma would be equal to *C*_FT_. A demonstration that the *V*_T_ for a compound is approximated by the ratio *f*_P_/*f*_ND_ provides confidence in drug transport by passive diffusion and hence the estimation of *C*_FT_ from peripheral plasma data.

We did not see conclusive evidence for a change in regional *V*_T_ values for GSK103702 following a pharmacological dose that was statistically significant, suggesting that any specific binding component of [^11^C]GSK1034702 is negligible compared to the free and non-specific components. The whole brain *V*_T_ in human was 4.9, which is broadly comparable to the measured *f*_P_/*f*_ND_ of 2.63, implying passive diffusion across the BBB. The difference between *V*_T_ and *f*_P_/*f*_ND_ is most likely due to experimental noise in the PET and equilibrium dialysis assays, but even if this difference represents a deviation from passive diffusion across the BBB, this deviation would be in the direction of an active transport of the molecule into the brain. Such a situation, were it to be clinically relevant would lead to a higher brain C_FT_ than the plasma-free concentration, and thus would minimise the incidence of peripheral side effects for a given central m_1_AChR occupancy.

Similarly, there was significant uptake of GSK1034702 into the brain at tracer quantities and following oral dosing of 5 mg in the human study. As there was some regional heterogeneity in the binding of GSK1034702 in the human brain, the cortex was used to derive a *V*_T_ assumed to be the most representative of the *V*_ND_, as this region produced *V*_T_ in the lower range of those examined. The whole brain *V*_T_ was 4.9, which is in broad agreement with primate *V*_T_ and the *f*_P_/*f*_ND_ ratio (3.97 and 2.63, respectively).

The results are broadly consistent across species, although the primate and human estimates are somewhat higher than the *f*_P_/*f*_ND_ ratio. Thus, if active transport plays a role in the BBB passage of GSK1034702, it would appear to be an active influx into the brain (leading to *V*_T_ > *f*_P_/*f*_ND_), rather than an extrusion of the compound from the brain (which would lead to *V*_T_ < *f*_P_/*f*_ND_). Thus, if anything, therapeutic levels of m_1_AChR occupancy may be achieved with even lower plasma concentrations and hence lower incidence of adverse events, than those in the passive diffusion scenario. The presence of an active transport mechanism would make extrapolation from tracer studies to pharmacological doses more problematic, due to possible changes in the transport kinetics at higher doses (e.g. due to saturation of the transporters). However, we do not think that this is an issue for GSK1034702, as our examination of a therapeutically relevant dose (5 mg p.o.) produced results similar to those at tracer dose. The inclusion of a therapeutic dose requires extra safety information and complicates the study design, but may be worth including in situations where active transport is suspected.

A regionally heterogeneous distribution of [^11^C]GSK1034702 was observed in humans, with the highest *V*_T_ observed in the medial temporal lobe (MTL), consistent with the known distribution of m_1_AChR in the human brain [16]. This finding is supported by a small (but non-significant) reduction in the MTL *V*_T_ following the administration of a 5-mg oral dose of GSK1034702, in both subjects for whom pre- and post-dose data was available. Our data are consistent with a small specific binding component for [^11^C]GSK1034702 in the human brain. The data from humans are in contrast to those in the baboon brain, where homogenous distribution was observed. A potential explanation for the between species differences in distribution could be a masking of high affinity states of m_1_AChR by ketamine [17,18] in the baboon brain. However, further studies would be needed to properly understand these differences.

The magnitude of the specific component may be too low for [^11^C]GSK1034702 to be a useful PET ligand for the m_1_AChR. This may be due either to a relatively low *in vivo* affinity of [^11^C]GSK1034702 for the m_1_AChR in relation to the target density or due to a relatively low specific activity (SA) and hence a high mass of GSK1034702, leading to partial blockade of the specific binding component being used in this study. The radiolabelling method used precludes the achievement of higher SA than reported here, and hence, [^11^C]GSK1034702 may not be suitable for the quantification of m_1_AChR density with PET. However, it may provide a useful lead in the search for related compounds with higher affinity for this target or compounds that may be labelled using methods leading to higher SA.

This study type has several limitations. For the PET data, the presence of brain-penetrant metabolites cannot be ruled out for all compounds using these methods. In this case, it is unlikely because there was little metabolism of the compound and because the data was well described by a two-tissue compartment model. For the equilibrium dialysis data, the duration of dialysis, temperature and buffer conditions and the fact that different primate brains were used for the *in vitro* and *in vivo* data of the primate study, as well as primate brain instead of human brain tissue for the human study, could all introduce errors into the measurements.

## Conclusions

In primate and human PET studies designed to evaluate the transport across the BBB of a novel selective M1 receptor allosteric agonist GSK1034702, we have demonstrated good brain uptake. Although there was variability in this limited dataset, BBB kinetics were consistent with passive diffusion or active influx.

The single oral dose of 5 mg GSK1034702 was well tolerated by healthy subjects in this PET study, and the pharmacokinetic parameters were consistent with those observed in a separate First-Time-In-Human safety and tolerability study (GSK study number 110623 and study number NCT00743405 on CT.gov).

In conclusion, an examination of the BBB kinetics of GSK1034702 in phase 0 and phase I of development has discharged some of the perceived development risks for GSK1034702 and provided information to progress the molecule into the next stage of clinical development.

## Additional files

Additional file 1: Figure S1A. Methods definition for hand drawn region of interest. Additional file 2: Table S1A. Supplementary table showing Regional K_1_ Values in Papio Anubis Brain.

## Abbreviations

M_1_: muscarinic-1
C: carbon-11
m_1_AChR: muscarinic acetylcholine receptor subtype 1
PET: positron emission tomography
CNS: central nervous system
BBB: blood-brain barrier
*V*_T_: volume of distribution
*C*_FT_: ‘free’ or unbound concentration of the drug in the tissue of interest
*K*_D_: affinity of the drug for its target
*C*_ND_: total non-displaceable tissue concentration
*f*_ND_: ‘free’ or unbound fraction
*C*_FP_: ‘free’ concentration of the drug in plasma
Occ_T_: fractional occupancy of the target
mAChR: muscarinic acetylcholine receptor
*C*_T_: total brain concentration of the drug
AD: alzheimer's disease
SZ: schizophrenia
PGP: P-glycoprotein
IACUC: Columbia University Institutional Animal Care and Use Committee
IV: intravenous
FWHM: full width at half maximum
ROIs: regions of interest
MRI: magnetic resonance imaging
*F*_P_: free fraction of drug in arterial plasma
SD: standard deviation
p.o.: orally
HR: high resolution
2D: two-dimensional
TACs: brain time activity curves
BMI: body mass index
HMR: HAMMERSMITH Medicines Research
3D: three-dimensional
CT: computed tomography
MTL: medial temporal lobe
*K*_1_: delivery rate constant for transfer from arterial plasma to tissue
PK: pharmacokinetic
SD: standard deviation
*V*_S_: concentration of drug specifically bound to the target
*V*_ND_: concentration of non-displaceably bound drug
