# Supplementary material for: An evaluation of the brain distribution of [11C]GSK1034702, a muscarinic-1 (M1) positive allosteric modulator in the living human brain using positron emission tomography
Source: EJNMMI Res. 2014 Dec 5;4:66. doi: 10.1186/s13550-014-0066-y (PMC4452589; doi:10.1186/s13550-014-0066-y)
Supplement: Additional file 1: Figure S1A. — Methods definition for hand drawn region of interest. [file 13550_2014_66_MOESM1_ESM.doc]

*Additional file 1*

*Hand drawn region of Interest (ROI) definition*

Regions of interest for this study were mainly defined using a standardised anatomical template. A heterogeneous binding was observed in the subject’s baseline images with the highest signal coming from striatum, thalamus, insula, hippocampus and the pituitary gland. In order to assess whether there was a specific binding signal individually defined regions of interest (ROI’s) were created for a subsection of the medial temporal lobe (MTL) within the hippocampus. In hippocampus a high signal was observed in the dorsal part of hippocampus’s head and tail. However, data suggested that the signal located around the hippocampus tail is from the choroid plexus and not from the hippocampus therefore the regional analysis was performed only for the hippocampus head. For the region of interest (ROI) analysis a set of fixed size ROIs were placed on the PET integral image and were subsequently applied to the dynamic PET data to derive regional time activity curves (TACs). The ROI is a 6 x 6 mm square and was placed on six successive coronal slices on a 2×2×2 (in mm) PET image. See Figure A to view a representative MTL ROI.


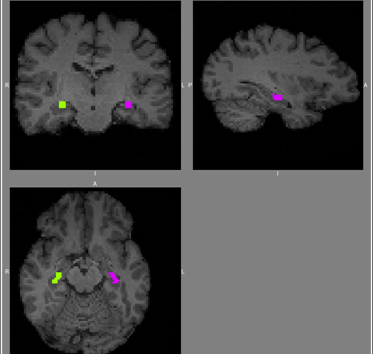

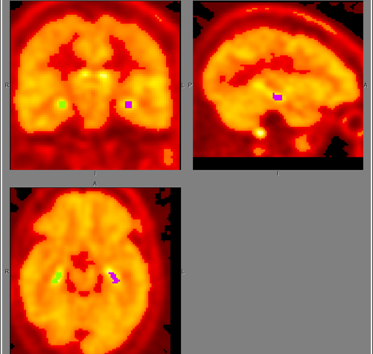


**Figure S1A. Example of hand defined region of interest (ROI) in the medial temporal lobe (MTL).** The regions of interest were mainly defined using a template with standard anatomical regions. There was an area of high signal in a subset of the hippocampus, in order to accurately assess this region an additional ROI was manually defined for each subject in this region. The image on the left shows the MTL ROI on the subjects MRI scan, the image on the left shows the same ROI on the co-registered PET image.
