# Supplementary material for: An evaluation of the brain distribution of [11C]GSK1034702, a muscarinic-1 (M1) positive allosteric modulator in the living human brain using positron emission tomography
Source: EJNMMI Res. 2014 Dec 5;4:66. doi: 10.1186/s13550-014-0066-y (PMC4452589; doi:10.1186/s13550-014-0066-y)
Supplement: Additional file 2: Table S1A. — Supplementary table showing Regional K1 Values in Papio Anubis Brain. [file 13550_2014_66_MOESM2_ESM.doc]

Additional file 2

**Table S1A. Regional K_1_ Values in Papio Anubis Brain**

| ROI | Subject A  Exam 1 | Subject A  Exam 2 | Subject B  Exam 1 |
| --- | --- | --- | --- |
| Cerebellum | 0.46 | 0.57 | 0.35 |
| Hippocampus | 0.39 | 0.60 | 0.32 |
| Thalamus | 0.44 | 0.52 | 0.27 |
| Striatum | 0.39 | 0.51 | 0.39 |
| Frontal Ctx | 0.39 | 0.53 | 0.32 |
| Occipital Ctx | 0.67 | 0.53 | 0.37 |
| Whole Brain | 0.45 | 0.50 | 0.35 |

K_1_ = Rate constant for transfer from arterial plasma to tissue (mL.cm^-3^.min^-1^)
